# Comparing public and private providers: a scoping review of hospital services in Europe

**DOI:** 10.1186/s12913-018-2953-9

**Published:** 2018-02-27

**Authors:** Liina-Kaisa Tynkkynen, Karsten Vrangbæk

**Affiliations:** 1 Faculty of Social Sciences, University of Tampere, FI-33100 Tampere, Finland; 2 Department of Public Health, University of Copenhagen, P.O. Box 2099, DK-1014 Copenhagen, Denmark

**Keywords:** Healthcare, Hospitals, Private providers, Not-for-profit providers, For-profit providers, Europe, Specialized care services, Scoping review

## Abstract

**Background:**

What is common to many healthcare systems is a discussion about the optimal balance between public and private provision. This paper provides a scoping review of research comparing the performance of public and private hospitals in Europe. The purpose is to summarize and compare research findings and to generate questions for further studies.

**Methods:**

The review was based on a methodological approach inspired by the British EPPI-Centre’s methodology. This review was broader than review methodologies used by Cochrane and Campbell and included a wider range of methodological designs. The literature search was performed using PubMed, EconLit and Web of Science databases. The search was limited to papers published from 2006 to 2016. The initial searches resulted in 480 studies. The final sample was 24 papers. Of those, 17 discussed economic effects, and seven studies addressed quality.

**Results:**

Our review of the 17 studies representing more than 5500 hospitals across Europe showed that public hospitals are most frequently reported as having the best economic performance compared to private not-for-profit (PNFP) and private for-profit (PFP) hospitals. PNFP hospitals are second, while PFP hospitals are least frequently reported as superior. However, a sizeable number of studies did not find significant differences. In terms of quality, the results are mixed, and it is not possible to draw clear conclusions about the superiority of an ownership type. A few studies analyzed patient selection. They indicated that public hospitals tend to treat patients who are slightly older and have lower socioeconomic status, riskier lifestyles and higher levels of co-morbidity and complications than patients treated in private hospitals.

**Conclusions:**

The paper points to shortcomings in the available studies and argues that future studies are needed to investigate the relationship between contextual circumstances and performance. A big weakness in many studies addressing economic effects is the failure to control for quality and other operational dimensions, which may have influenced the results. This weakness should also be addressed in future comparative studies.

## Background

Public funding, as well as public provision of healthcare services, has been a key feature of many modern welfare states. However, since the 1980s the realms of the public and private sectors have been redefined in many countries [43]. At the same time, systems financed through social or private insurance have developed new ways of organizing their relationships with providers. What is common to all healthcare systems is a discussion about the optimal balance between public and private provision.

In a seminal paper from 1963, Kenneth Arrow demonstrated that health care has a number of characteristics that violate the principles of a perfect market [3]. Healthcare consumers do not have sufficient information to know when and to what extent health care is needed or to compare alternatives. Externalities are not incorporated in decision making, and patients risk catastrophic losses in the event of serious illness. Attempts to solve this problem through private insurance carry other risks in terms of adverse selection and moral hazards. As a consequence, all modern healthcare systems have some degree of public involvement in the regulation, financing or provision of services. The implication is that health care is delivered in highly regulated markets with different combinations of public and private actors [7]. This leads us to ask whether there is evidence that private delivery organizations perform better than public delivery organizations in regulated health care markets.

We investigated this question by conducting a scoping review of the available evidence from recent studies within the European region. Although this region includes different types of healthcare systems, all countries rely considerably on public or not-for-profit providers in addition to some degree of private for-profit delivery. Focusing on the European region allowed us to include systems that are based on similar values about solidarity, while excluding studies from countries with radically different underlying values, such as the United States (US) and Singapore. At the same time, by including the entire region, we can expand on the degree of diversity and volume compared to previous studies, such as Tiemann et al. [50].

Our method was a scoping review which aimed to summarize and compare previous studies presenting evidence on differences in performance between public and private hospitals in European healthcare systems. Scoping reviews aim to “map rapidly the key concepts underpinning a research area and the main sources and types of evidence available and can be undertaken as stand-alone projects in their own right” [2]. The specific purpose of this review was to summarize and compare research findings, to relate the findings to previous reviews and to generate questions for further studies and systematic reviews.

### Theoretical perspectives on public–private comparisons

Theoretical claims for positive effects of private ownership typically stem from public choice and property rights theories, which revolve around a competition and a public management/ownership argument, respectively [1, 13, 21]. The competition argument states that although healthcare markets may be imperfect, competition in itself can have beneficial effects. Private providers are forced by competitive pressure to optimize efficiency, while political and administrative pressures are more important for public providers. The lack of competitive pressures means that public managers are unable to measure the efficiency of their organizations against a commercial bottom line. Decisions on resource allocation and survival of the organization are left to public decision makers who cannot rely on market prices to generate an equilibrium between demand and supply.

The public management/ownership argument states that public sector organizations lack incentives to perform efficiently, these organizations often have broad and conflicting objectives, and they have no bankruptcy constraint. That is, they can continue to perform at sub-optimal levels without the risk of going out of business [1]. Furthermore, public organizations are not accountable to shareholders and owners and therefore, potentially have less external pressure to focus on innovation and technological development. Finally, it has been argued that a major difference between public and private hospitals is that public hospitals tend to operate in settings with “soft budget constraints” [22, 40]. Some countries have tried to overcome this difference through various types of purchaser–provider splits [7] and legislation regarding hard budget constraints such as the Danish “Budget Law.”

Several theoretical contributions have nuanced and broadened the expectations from public choice and property rights theory [10, 53]. Transaction cost economics emphasizes the importance of asset specificity and the measurability of the services that are provided in the market [15, 54]. Rather than approaching public services as something that would, by definition, be more effectively produced in a private market, transaction cost economics hypothesizes that different service characteristics create more or less favorable conditions for in-house production and contracting [29]. Economic benefits from contracting are more likely to be realized if the quantity and quality of the services can be unambiguously described and measured. Otherwise, the costs of preparing tenders, evaluating bids, signing contracts and monitoring (and possibly sanctioning) service delivery are likely to be high. The largest economic effects, thus, are expected in technical services characterized by low asset specificity and high measurability, whereas smaller or even negative economic effects would be expected in complex services with high asset specificity and low measurability. For hospitals, this would lead us to expect that standardized procedures, for example, within some surgical areas and technical support functions are more likely to provide privatization benefits than complex services within the field of psychiatry or geriatrics, for instance. Hospitals are complex organizations, which typically include high- and low-specificity services. According to asset specificity theory, this leads to additional uncertainty about the benefits of privatization compared to the competition and ownership argument.

Industrial organization theory stresses a number of factors that make public markets distinct from traditional private markets and thus, create less optimal conditions for contracting out than expected by public choice theory [10]. According to this perspective, many public services are characterized by natural monopolies and high entrance costs, which limit competition and potentially make highly regulated markets with public providers less efficient than private markets [26]. Principal-agent theory further emphasizes the problem of information particularly in markets for welfare services, such as health, social and child care, where those buying the service have limited insight into the actual delivery practice of the agents. The presence of information asymmetries can lead to goal displacement and unwanted practices, such as “cream-skimming” (selection of the easiest tasks) and “parking” of the least profitable clients. This can endanger the system-level benefits assumed in perfect market conditions.

Decreasing marginal effects from contracting out suggests that economic effects tend to decrease over time [8, 34, 35]. There are two theoretical claims behind this argument. First, it is likely that rational purchasing organizations begin with contracting out those services and tasks where the largest gains are expected. Once the organizations have harvested the low hanging fruits, we can expect decreasing benefits from additional contracting out [9, 34]. Second, involvement of private providers creates competitive pressure on public in-house production units, which may lead to more effective public production [5]. The market mechanism and exposure to competition, according to this argument, increase the efficiency of not only the contracted services but also the internally produced services [9]. Once the public providers have adjusted their operational practices, there will be few or no additional gains from switching to private providers.

The focus of this paper was to provide an empirical overview of efficiency results as reported in the empirical studies we identified in our database searches. The studies employed slightly different definitions and techniques (see Table 3), but data envelopment analysis (DEA) and stochastic frontiers analysis (SFA) techniques dominate. Technical and allocative efficiency comprises “overall efficiency” [33]. Technical efficiency is producing the maximum amount of output from a given amount of input or alternatively, producing a given output with minimum input quantities, such that when an organization is technically efficient, it operates on its production frontier. Allocative efficiency occurs when the input mix is that which minimizes cost, given input prices or alternatively, when the output mix is that which maximizes revenue, given output prices.

In addition to efficiency differences, we reviewed evidence of potential quality differences and operational differences between public and privately owned organizations. Operational differences include factors such as patient selection, staff composition and procedures that may include thresholds for admissions. In terms of quality, the measurements used were diverse which made it difficult to draw clear conclusions across the studies. Still, quality and operational parameters are important as they relate to other policy objectives than efficiency. However, very few studies embarked on multidimensional assessments, and narrow efficiency measures were, by far, the most commonly reported dimension.

### Setting the stage: The results from previous review studies

We start by summarizing state-of-the-art as presented in previous international review papers that examined differences in economic and/or quality performance between private and public hospital organizations. The review studies were not included in the core sample, as we focused on primary studies published from 2006 to 2016 within the European region. Herrera et al. [32] provided an overview of systematic reviews of the performance of private for-profit (PFP), private not-for-profit (PNFP) and public healthcare providers. The authors reviewed 5918 references to identify systematic reviews and ended up with nine relevant studies of sufficiently high quality. According to the nine systematic reviews, ownership appears to have an effect on health- and healthcare-related outcomes. In the comparison of PFP and PNFP providers, significant differences in terms of patient mortality and payments to facilities were found; both were higher in PFP facilities. In terms of quality and economic indicators, such as efficiency, there were no significant results. When PNFP and public providers were compared, as well as PFP and public providers, no clear differences were found. The overall conclusion from the study was that PFP providers seem to have poorer results than their PNFP counterparts, but there are still important evidence gaps in the literature that need to be covered.

Currie et al. [18] reviewed 34 studies. Most of these studies found no difference between PFP and PNFP full-service hospitals in terms of relative costs, quality of care or efficiency. Shen et al. [46] employed a quantitative method when reviewing 40 studies to identify the factors that explain the different findings for cost, revenue, profit margin and efficiency in the empirical literature. The authors found that variations in the magnitudes of ownership effects could be explained by the research focus and methodology of the individual studies. Studies using empirical methods that controlled for a few confounding factors tended to find larger differences between PFP and PNFP hospitals than studies that controlled for a wider range of confounding factors. Functional form and sample size also matter. Failure to apply log transformation to highly skewed expenditure data yielded misleadingly large estimated differences between PFP hospitals and PNFP hospitals. Studies with fewer than 200 observations also produced larger point estimates and wider confidence intervals. In a follow-up study conducted in 2008 by Egglestone et al., the authors found that pooled estimates of ownership effects are sensitive to the subset of studies included and the extent of overlap among hospitals analyzed in the underlying studies [23]. Ownership appears to be systematically related to differences in quality among hospitals in several contexts. Whether studies found PFP and public hospitals have higher mortality rates or rates of adverse events than their PNFP counterparts depended on the data sources, time period and region covered.

Tiemann et al. [50] investigated hospital ownership and efficiency in a review of studies that focused on Germany. The authors concluded that in line with the evidence found in studies from other countries, especially the US, the evidence from Germany suggests that private ownership (i.e., PFP and PNFP) is not necessarily associated with higher efficiency compared to public ownership. Irvin’s [36] review of studies of U.S. healthcare organizations showed that there is a quality gap between for-profit and nonprofit firms in some healthcare sectors (long-term care and mental health), depending on the prevailing type of financial payment for health care.

Hollingsworth [33] reviewed 317 studies published until 2006. He concluded cautiously “that public provision may be potentially more efficient than private, in certain settings.”

The overall impression from previous review studies is mixed. Some studies found that public hospitals are more efficient than private, while others found no significant difference. In general, it appears that PNFP hospitals tend to be closer to public hospitals in outperforming PFP hospitals in terms of quality and efficiency.

These diverging and somewhat surprising results inspired two groups of scholars [23, 46]) to investigate the methodological basis for the results. The authors emphasized that case selection, methodological approach, time period and region are important underlying factors. A general observation across the studies was that the true effect of ownership seems to depend on the institutional context and that there are significant differences across regions and markets and over time.

## Methods

The aim of this paper was to add an update to the results described above. We do that by providing a scoping review of peer-reviewed primary studies on public–private comparisons in specialized health care. We focused on studies that were conducted over the past decade within the European region.

Scoping reviews aim to “map rapidly the key concepts underpinning a research area and the main sources and types of evidence available and can be undertaken as stand-alone projects in their own right [2]. These reviews can typically have any of four motivations: (1) to “examine the extent, range and nature of research activity,” that is, a mapping to elucidate the extent and range of research in the area; (2) “to determine the value of undertaking a full systematic review”; (3) to “summarize and disseminate research findings”, operating in the direction of a systematic review, describing findings in greater detail and acting to summarize and disseminate findings to key stakeholder audiences with the intention of informing those stakeholders and eliminating or reducing the need to undertake a more in-depth review; and (4) to “identify research gaps in the existing literature.” In our case, we aimed to summarize research findings and generate questions for further studies and systematic reviews.

The review was based on a methodical approach inspired by the British EPPI-Centre’s methodology. This review was broader than review methodologies used within the Cochrane and Campbell collaborations, which emphasized randomized controlled trials (RCTs) as the gold standard [38]. The present review also included a broader range of methodological designs and quantitative and qualitative studies Petersen et al. [42].

The literature search was conducted using PubMed, EconLit and Web of Science databases. The search was limited to papers published from 2006 to 2016. The limitation to the most recent decade was to avoid too much overlap with previous reviews while including the most recent studies. The inclusion criteria were papers written in English that dealt with the European region. The search strategies for the databases are presented in Table 1.

**Table 1:** Search strategies and databases

Database	Search strategy
PubMed (354 hits)	((“ownership”[MeSH Terms] OR “ownership”[All Fields]) OR (“contracts”[MeSH Terms] OR “contracts”[All Fields] OR “contracting”[All Fields]) OR (“outsourced services”[MeSH Terms] OR (“outsourced”[All Fields] AND “services”[All Fields]) OR “outsourced services”[All Fields] OR “outsourcing”[All Fields]) OR bidding[All Fields]) AND (public[All Fields] AND (“patients’ rooms”[MeSH Terms] OR (“patients’“[All Fields] AND “rooms”[All Fields]) OR “patients’ rooms”[All Fields] OR “private”[All Fields])) AND ((“economics”[Subheading] OR “economics”[All Fields] OR “cost”[All Fields] OR “costs and cost analysis”[MeSH Terms] OR (“costs”[All Fields] AND “cost”[All Fields] AND “analysis”[All Fields]) OR “costs and cost analysis”[All Fields]) OR saving[All Fields] OR quality[All Fields] OR (“efficiency”[MeSH Terms] OR “efficiency”[All Fields])) AND (“hospitals”[MeSH Terms] OR “hospitals”[All Fields] OR “hospital”[All Fields]) AND (“2006/09/11”[PDat]: “2016/09/07”[PDat] AND English[lang])
EconLit (93 hits)	(hospital* AND (ownership OR contracting OR outsourc* OR bid*) AND (cost* OR saving* OR quality OR efficiency))
Web of Science (53 hits)	(((hospital) AND (ownership OR contracting OR outsourcing OR bidding) AND (public AND private) AND (cost OR saving OR quality OR efficiency))) Refined by: LANGUAGES: (ENGLISH) AND DOCUMENT TYPES: (ARTICLE) AND COUNTRIES/TERRITORIES: (GREECE OR ENGLAND OR GERMANY OR NETHERLANDS OR NORWAY OR ITALY OR DENMARK OR SWEDEN OR FINLAND OR SCOTLAND OR FRANCE OR WALES OR CZECH REPUBLIC OR BELGIUM OR CROATIA OR SLOVAKIA OR AUSTRIA) Timespan: 2006–2016. Indexes: SCI-EXPANDED, SSCI, A&HCI, ESCI.

The assessment and compilation of the final sample of relevant studies included three phases. Phase 1 included a search for relevant literature. The initial searches resulted in 480 studies: 354 from PubMed, 93 from EconLit and 53 from Web of Science of which some were duplicates. In phase 2, the abstracts were sorted using the categories not relevant, perhaps relevant and relevant. The not relevant category included papers that were not based in Europe or in which public–private comparisons were not found. The perhaps relevant category included papers whose suitability could not be judged solely on the abstract. Phase 3 included the final assessment of the relevance of the papers. For the relevant or perhaps relevant abstracts, the full papers were further examined, which resulted in grouping the studies that were finally included in the study and studies that were found not relevant after the full paper was read. In this phase, the not relevant papers were mostly theoretical papers, papers in which there were, eventually, no empirical public–private comparisons or very vague descriptions of the comparative material. At this stage of the process, we also excluded studies that addressed outsourcing, privatization and corporatization of hospitals with a focus on the dynamic process of transfer from one ownership type to another.

**Fig. 1:**
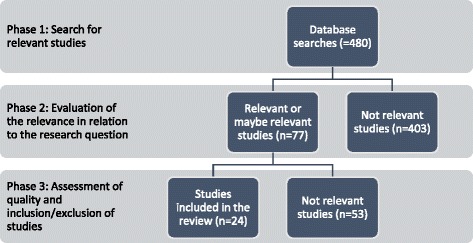
Overview of the review procedure

The final sample of studies that fulfilled the inclusion criteria was 24 papers. All of the papers were published in peer-reviewed journals, and we did not conduct further quality evaluations as the papers had undergone a peer-review process (Fig. 1).

**Fig. 2:**
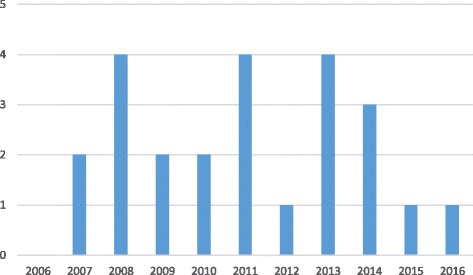
Number of studies by year

The studies represented 10 countries (Table 2). Since 2006, we observed a slight increase in the number of papers published on the subject (Fig. 2). This increase confirms the trend observed by Hollingsworth although he reported a “dramatic” increase over the past decades [33].

**Table 2:** Number of studies by country

	Austria	Denmark	England	France	Germany	Greece	Italy	Norway	Spain	Switzerland
No of studies	1	3	2	1	6	2	5	1	1	2

Most often, the studies in this sample involved comparisons of two groups: public and private hospitals (*n* = 13). However, the definitions of public and private varied. Eleven studies made clear distinctions between public, PFP and PNFP hospitals. Economic effects were explored in 17 studies and quality in seven studies (in three studies, it was used as a control for economic effects). Patient selection was mentioned in 15 studies but discussed explicitly in only seven studies.

## Results

The majority of the studies (*n* = 17) found in the database searches addressed the economic performance of public and private specialized care organizations. Seven studies addressed quality.

In terms of economic performance, 15 studies compared public (PUB) hospitals to PFP hospitals. Some studies reported technical, cost and profit efficiency (see Table 3). About half of these studies reported that public hospitals are superior to PFP hospitals in terms of efficiency. Most of the other studies found insignificant differences. Only one study reported that PFP hospitals have better profit efficiency. Eight studies compared the performance of PFP and PNFP hospitals. The majority of these studies found that PNFP hospitals are superior in terms of technical, cost and profit efficiency. Only one study pointed to responsiveness as a performance measure where PFP hospitals are better than PNFP hospitals. Finally, we found 11 studies compared PUB and PNFP hospitals. Most of these studies reported insignificant differences. In the remaining studies, we found slightly more studies presented PUB hospitals as superior to PNFP hospitals.

**Table 3:** Empirical studies reporting economic effects

	Study and sample characteristics			Methods and control variables					Difference between ownership type (better is indicated)^a^		
	Country	Years	Sample size	Main focus	Patient heterogeneity	Hospital characteristics	Market or environmental characteristics^b^	Quality of care	PB vs. PFP	PB vs. PNFP	PFP vs. PNFP
Caballer-Tarazona et al. [17]	Spain	2009–2010	29 hospitals	DEA^c^ - Efficiency	X	X			n.s.		
Bonastre et al. [14]	France	2007–2008	448 hospitals	The use of expensive anticancer drug		X			n.s	–	–
Czypionka et al. [19]	Austria	2010	128 hospitals	DEA – technical efficiency	X	X	X		–	PNFP	–
Siciliani et al. [45]	England	2006–2007	193 hospitals or clinics	Length of stay	X	X	X		PFP^d^		
Augurzky et al. [4]	Germany	2001–2005	331 hospitals	Probability of default		X	X		PFP	PNFP	PNFP
Kondilis et al. [37]	Greece	2001–2003	320–330 hospitals	The operation and performance: 1) nurse staffing rates 2) ALoS 3) SHI^e^ payments for hospital care per patient discharged.		X			PB^perf,f^ PB^payments,g^	–	–
Herr et al. [31]	Germany	2002–2006	541 hospitals or small chains	SFA^h^- Technical, cost and profit efficiency	X	X	X	x	n.s ^tech^ n.s ^cost^ PFP^profit^	n.s. ^tech^ n.s. ^cost^ PNFP^profit^	–
Schwierz [49]	Germany	1996–2006	16,356,428 patient admissions to 1817 hospitals (2006) and 14,921,393 patient admissions to 2040 hospitals (1996)	Responsiveness to changes in demand for hospital services	X		x		PFP	n.s.	FP
Berta et al. [12]	Italy	1998–2007	The full population of patients and hospitals operating in Lombardy, c. 20,000,000 admissions	DEA - Technical efficiency	X	X			PB	n.s.	NP
Kontodimo-poulos et al. [39]	Greece	2004	124 dialysis facilities	DEA – technical efficiency		X	X		n.s.		
Daidone and D’Amico [20]	Italy	2000–2005	108 hospitals	SFA - Technical and cost efficiency	X	X	X		PB	PB	PNFP
Tiemann et al. [51]	Germany	2002–2006	1046	DEA – Technical efficiency	X	X	X	X	PB	PB	n.s.
Herr [30]	Germany	2000–2003	1556–1635 hospitals	SFA - cost and technical efficiency	X	X		X	PB	PB	PNFP
Farsi and Filippini [25]	Switzerland	1998–2003	148 hospitals	SFA - cost efficiency (ownership and different subsidy types)		X			n.s.	n.s.	n.s.
Farsi [24]	Switzerland	1998–2002	214 hospitals	SFA - cost efficiency (ownership and different subsidy types)		X			n.s.	n.s.	n.s.
Berry et al. [11]	Germany	2002–2003	89 hospitals	Operating room productivity		X	X		n.s		
Barbetta et al. [6]	Italy	1995–2000	531 hospitals	DEA, SFA, COLS^i^ - technical efficiency in two periods: 1995–1997 before DRG^j^ payment system introduction 1998–2000 after DRG payment system introduction		X				NP^1995–1997^ n.s.^1998–2000^	

^a^*PB* public, *PFP* private for-profit, *PNFP* private not-for-profit

^b^Environmental characteristics included here

^c^*DEA* data envelop analysis

^d^When private specialized treatment center (STCs) are compared with public hospitals. In addition, public STCs did better than hospitals, but private ones outperformed them as well

^e^*SHI* Social Health Insurance

^f^PFP hospitals have lower bed capacity, lower occupancy rates and lower nurse (total and high qualified) staffing rates compared to public hospitals and are associated with higher unweighted length of stay

^g^PFP hospitals have higher SHI payments per discharge

^h^*SFA* stochastic frontiers analysis

^i^COLS corrected ordinary least squares

^j^DRG diagnosis related group

Overall, it seems that in terms of economic performance the public hospitals in the 17 studies representing more than 5500 hospitals across Europe perform better than PNFP hospitals, which, in turn, perform better than PFP hospitals. However, a sizeable number of studies did not find significant differences. In terms of quality, the results were mixed, and it is not possible to draw clear conclusions about the superiority of an ownership type.

The following sections provide details about the studies and their results.

### Economic performance: Technical, cost and profit efficiency

Berry et al. [11] looked at operating room productivity in independent anesthesiology departments within German hospitals by using survey data from 87 hospitals. The authors hypothesized that operating room productivity is higher for hospitals run by private corporations compared to those run by the public sector. In the analysis, they found some confirmation of this idea but presented no significant results. The overall conclusion was that hospital size is the single largest predictor of productivity. However, the authors also suggested that micro-level management processes matter.

Kontodimopoulos et al. [39] found that after controlling for contextual characteristics technical efficiency was not significantly different between public and private dialysis facilities in Greece. The authors concluded that the context rather than ownership influences the performance of service providers. Barbetta et al. [6] stressed the importance of contextual factors and reimbursement practices in a study in which they looked at the technical efficiency of public and PNFP hospitals in Italy. The authors suggested that the differences in economic performance are related to institutional settings in which providers operate rather than to the ownership per se.

Czypionka et al. [19] looked at the impact of ownership on efficiency in Austria. Contrary to several previous studies, the authors found that there is a significant association between efficiency and ownership when comparing public and PNFP hospitals. The latter outperform public hospitals in technical efficiency due to different financial incentives.

Herr [30] found that in Germany PFP and PNFP hospitals are, on average, less cost-efficient and less technically efficient than publicly owned hospitals. This result can be partly explained by the importance of length of stay, which was, at the time, highest in PFP hospitals. Similar results were found in the study by Tiemann and Schreyögg [51] who evaluated the efficiency of public, PFP and PNFP hospitals in Germany. The results showed that public hospitals perform significantly better than PFP and PNFP hospitals. However, Herr et al. [31] found no significant differences in cost and profit efficiency between public and PFP hospitals in Germany.

Daidone and D’Amico [20] looked at how the production structure and level of specialization of a hospital affect its technical efficiency in Italy. They found that PFP hospitals use resources less efficiently compared to public and PNFP hospitals. PFP hospitals work in slightly over-staffed conditions for medical staff while public and especially PNFP hospitals are over-staffed by technical and administrative staff. Caballer-Tarazona et al. [17] compared public hospitals and public–private partnership (PPP) model hospitals in the Valencia region, but they were not able to determine the effect of ownership on efficiency due to the small sample size.

### Comparisons of costs and other economic outcomes

Two studies—both from Switzerland employing similar data—found that hospital ownership does not affect hospital costs [24, 25]. Bonastre et al. [14] analyzed the use of expensive anticancer drugs in public and private hospitals. The authors found that there were significant differences in terms of capacity, volume of activity and case mix between private and public hospitals, but after adjusting for the case mix, there were no differences in the use of expensive drugs between private and public hospitals.

Kondilis et al. [37] compared the operation and performance of PFP and public hospitals in Greece, focusing on differences in nurse staffing rates, average lengths of stay and Social Health Insurance (SHI) payments (including per diem fees, plus additional fee-for-service payments for services provided during hospitalization) for hospital care per patient discharged. The authors found that there were differences between PFP and public providers operating within the mixed healthcare system. PFP hospitals had lower bed capacity, lower occupancy rates and lower nurse (total and high qualified) staffing rates compared to public hospitals. PFP hospitals are also associated with higher unweighted length of stay and higher payments per discharge, at least in the case of discharged patients are beneficiaries of the SHI funds.

Siciliani et al. [45], in turn, studied patients’ length of stay in public hospitals, specialized public treatment centers and private treatment centers that provide elective hip replacement in England. The authors found that public and private specialized treatment centers, on average, had 18% and 40% shorter lengths of stay, respectively, compared with public hospitals. The result remained the same after controlling for age, gender, diagnosis and market characteristics. They did not find that patient selection explains differences in the length of stay in different hospital settings.

Augurzky et al. [4] studied the differences between public, PFP and PNFP ownership types in German hospitals based on their probability of default (PD). According to the results, public hospitals tend to exhibit a PD that is significantly above average. This association indicates that public ownership may conflict with financial sustainability. The authors explained it by stating that it is possible that public guarantees are the key driver to explain the differences. Public backing opens the window that ceteris paribus public hospitals may have higher PDs without being necessarily closer to insolvency than private hospitals.

Schwierz [49] studied ownership-specific differences in the responsiveness of changes in demand for hospital services in Germany from 1996 to 2006. He found that in the speed of adaptation to increasing demand PFP ownership is superior to public and PNFP ownership. PFP providers also tend to expand in markets with decreasing demand. This result can be partly explained by the results found by Augurzky et al. [4] for higher probability of default. That is, the defaults of public hospitals nurture the process of privatization of public sector actors in a situation in which the public sector needs to reform their facilities and work practices while at the same time containing costs.

### Quality

Solborg Bjerrum et al. [47, 48] conducted two studies in Denmark that addressed the quality of elective surgeries in public and private hospitals. The 2015 study concerned patients who had cataract surgery in either public or private eye clinics or hospitals from 2002 to 2010. The results showed that patients who have cataract surgery in public hospitals have an overall statistically significant 62% higher mortality rate compared to patients who have cataract surgery in private hospitals or clinics. The potential explanation may be in the patient selection since the results indicate that patients who have cataract surgery in public hospitals are less healthy than patients who have cataract surgery in private hospitals or clinics (see more in the next section).

Another study by Solborg Bjerrum et al. [48] in Denmark addressed the risk of postoperative endophthalmitis (PE) in public and private eye clinics or hospitals from 2004 to 2012. The results showed that PE risk is 0.36 per 1000 operations in public hospitals and 0.73 per 1000 operations in private hospitals. Further analysis of the clinics revealed that there is homogeneity in the PE risk among the eye departments in public hospitals (*p* = 0.6) but heterogeneity in the PE risk among the private hospitals or eye clinics (*p* = 0.0001). Six private hospitals or clinics (out of 28) had a statistically significantly higher PE risk compared with the eye departments in public hospitals.

The third study from Denmark concerned how ownership affects professional behavior, treatment quality and patient satisfaction. In a mixed-methods study, Bøgh Andersen and Jakobsen [16] found that private clinics optimize non-clinical factors, such as wait times, more than public providers. The clinical procedures in the clinics, however, were very similar, and private clinics did not achieve better clinical results. Patient satisfaction was still higher in private clinics. Thus, the general conclusion of the study was that although ownership seems to influence certain aspects of care, the high level of professionalization neutralizes the effect which can be seen in the clinical results.

Pérotin et al. [41] studied whether hospital ownership affects the level of quality reported by patients in areas other than clinical quality (information and interpersonal care, respect for privacy, dignity and hospitality and delays) in England. The authors found that results vary across specialties and patient groups. The sum of all ownership effects was not statistically significant which led the authors to conclude that hospital ownership does not seem to determine the level of quality of the average patient’s reported experience. The authors also stated that the differences in the quality levels between the private and public sectors are mostly attributable to patient characteristics, patient selection into public or private hospitals and unobserved and specific hospital characteristics, rather than to hospital ownership.

Sanjay et al. [44] studied patient selection criteria, anesthetic preferences and outcomes of elective inguinal hernia repair in public and private sectors in England. The authors found that the mean wait time for patients undergoing hernia repair is 129 days in the public sector (range 16–379 days) and 15 days (range 8–61 days; *p* = 0.001) in the private sector. Caballer-Tarazona et al. [17] found some evidence that private ownership (PPP) seems to have a positive effect on some quality dimensions, such as access to care. In readmissions, Berta et al. [12] found that PNFP hospitals show the highest frequency of readmissions compared to public and PFP hospitals.

Sanjay et al.’s [44] results also showed differences in treatment practices: Anesthesia appears to be the preferred option in the private sector (52%) and local anesthesia in the public sector (66%; (*p* = 0.0002). After a follow-up at 6 months, there was a postal questionnaire survey regarding chronic groin pain and satisfaction rates. No statistically significant difference was noted in the incidence of post-operative complications, recurrence and groin pain and satisfaction rate between the patients treated in public or private facilities. Grilli et al. [27], in turn, found that ownership status and payment structure have a strong impact on the adoption and use of a new technology, drug-eluting stents. Public hospitals use drug-eluting stents more selectively than private hospitals targeting the new device at patients who have a high risk for adverse effects.

Grotle et al. [28] studied sociodemographic, lifestyle and clinical characteristics in patients who were operated for lumbar disc herniation in public and private clinics in Norway. The authors evaluated whether selection for surgery and surgical treatment differed between public and private clinics. The main results were that more patients operated in private clinics are sent home the same day of surgery, and a larger proportion of the patients receive prophylactic antibiotic treatment. There were also more complications in public clients compared to the private clinics. However, the patients treated in the private sector were different compared to the patients treated in the public clinics. This, again, may be the explanation behind the results. We turn to the discussion on patient selection in the following section.

### Operational differences

#### Patient selection

In terms of performance, it is relevant to assess whether hospitals engage in patient selection to reduce their risks and costs. In an unregulated competitive market, this may be a rational reaction, but it also creates a problematic bias in the results if the patient base varies significantly between public and private hospitals in individual studies.

Solborg Bjerrum et al. [47] found that patients treated in public and private settings are significantly different. The mean age at first eye cataract surgery decreased statistically significantly during the study period but significantly more so in patients operated in private hospitals or clinics than patients operated in public hospitals. Furthermore, the results of the mortality analyses indicated that patients who have cataract surgery in public hospitals are not as healthy as patients who have cataract surgery in private hospitals or clinics. Bøgh Andersen and Jakobsen [16] found that private hip replacement clinics have fewer complications than patients than public clinics.

Berta et al. [12] showed that private hospitals are involved in cream skimming at a much higher rate than public and not-for-profit hospitals. Sanjay et al. [44], in turn, found in England that patients undergoing surgery in the private sector are slightly younger compared to those treated in the public sector, that the number of patients with the American Society of Anesthesiologists (ASA) grading system grades III and IV is higher in the public sector (28.6%), and that there are a higher number of ASA I and II (83%) patients in the private sector.

In a study conducted in Italy, Grilli et al. [27] showed that patients in public hospitals are older and more likely to undergo percutaneous coronary intervention (PCI) for indications such as acute myocardial infraction and unstable angina than patients in private hospitals. In addition, patients with stable angina are more prevalent in private hospitals than in public hospitals. Furthermore, patients with multivessel disease who undergo PCI with stenting are significantly more prevalent in public centers with and without open-heart surgical facilities than in private centers. Finally, the proportion of patients with high-risk lesions is higher in public hospitals than in private hospitals.

Grotle et al. [28] found that patients who have lumbar disc herniation surgery in a private clinic are somewhat younger (1.3 years), are more likely to be male, have higher education and are less likely to be unemployed. The proportion of patients who were on sick leave was somewhat higher in private clinics than in the public sector. However, the duration of sick leave before surgery was significantly higher. In the public sector, the mean duration was 24 weeks (SD = 36.4) whereas in the private sector it was around 15 weeks (SD = 20.7). Grotle and colleagues also found that the proportions of disability and retired pensioners are more than double in the public sector compared to that for private clinics. There were also higher proportions of patients who smoked and were obese (BMI > 30) in the public health services. Furthermore, public sector patients used more pain relief, had a longer duration of pain in the back and leg, and had more comorbidities, such as heart disease, hip osteoarthritis, depression and chronic lung diseases. There was also a higher ASA grade among patients operated in public hospitals.

In sum, the limited number of studies analyzing patient selection indicated that public hospitals tend to treat patients who are older and have lower socioeconomic status, riskier lifestyles and higher levels of co-morbidity and complications than patients treated in private hospitals.

### Other operational dimensions

Other operational dimensions, such as differences in staff composition, skill level and working conditions, are very likely, but were not reported systematically in the studies included in this study sample. Berta et al. [12] analyzed effects of distortions (i.e., upcoding, cream skimming and readmissions) induced by the prospective payment system on hospitals’ technical efficiency in Italy. They found that PNFP and public hospitals have the same efficiency levels, while PFP hospitals have the lowest technical efficiency. This could be at least partially explained by the finding that private hospitals are more engaged with cream skimming which, in turn, was found to have a negative impact on hospitals’ technical efficiency. The role of the payment structure was also taken up by Augurzky et al. [4]. They found that public hospitals tend to exhibit PD at much higher levels than the hospitals in the sample did, on average. This could be explained by the public backing which affects hospital incentives to perform in a financially sustainable way (compare, e.g., [40]). Differences in financial incentives to hospitals of different ownership status were also brought up by Czypionka et al. [19] and Barbetta et al. [6], and both suggested that the different financial incentives are actually the key driver behind the different results in performance.

The study by Bøgh Andersen and Jakobsen [16] suggested that non-clinical practices, such as wait times, differ between public and private sectors, but in terms of clinical practices, organizations operate similarly. Kondilis et al. [37] found that PFP hospitals have lower bed capacity, lower occupancy rates and lower nurse staffing rates compared to public hospitals. Staffing rates were also discussed by Daidone and D’Amico [20] who found that PFP hospitals work in slightly over-staffed conditions for medical staff while public and especially PNFP hospitals are over-staffed by technical and administrative staff.

## Discussion

Numerous important theoretical contributions suggest that private hospitals should outperform public hospitals in terms of efficiency [19, 31, 52]. However, as we have seen, the empirical evidence from the regulated and mixed healthcare markets in Europe is much more diverse. Although many studies reported insignificant results, the majority of the remaining studies found that public hospitals perform better than PNFP providers, which, in turn, show slightly better performance than PFP hospitals in terms of efficiency measures (see Table 3). This result is in line with the conclusion in previous review studies, such as Hollingsworth [33] who summarized his findings as follows: “Cautious conclusions are that public provision may be potentially more efficient than private, in certain settings.” Tiemann et al. [50] concluded that in line with the evidence found in studies from other countries, especially the US, the evidence from Germany suggested that private ownership (i.e., PNFP and PFP) is not necessarily associated with higher efficiency compared to public ownership.

The last part of the Hollingsworth quote is important as it points to the discussion we launched in the introduction of this paper. Namely, that the context is important for understanding the results. Several studies discussed the specifics of the financing system, the contracting process and the degree of competition or monopoly in the market as important factors in determining the effects of ownership. In general terms, it appears likely that results are sensitive to specific circumstances and regulatory setup. Or as stated in one of the previous review studies,” [t]he true effect of ownership appears to depend on institutional context, including differences across regions, markets, and over time” [23].

Drawing on the theoretical contributions from the introduction, we speculate that variation in the results across countries and over time may be partially explained by differences in transaction costs, market structure and market maturity. High transaction costs may affect efficiency results for private providers more than for public providers, as administrative burdens may be internalized by public organizations. Market structure is a key issue as monopolies are likely to lead to lower efficiency, whether public or private. This means that diverging results across studies may be explained by underlying variations in market structure. Market maturity may also influence results across studies. As explained in the introduction, cost reductions tend to be highest in the first rounds of competitive bidding, while private and public agents adjust over time. Unfortunately, the studies did not report systematically on transaction costs, market structure or market maturity.

In terms of the ownership argument presented in the introduction, several countries operate with different types of private ownership, and PNFP organizations tend to do well in comparison with their PFP counterparts. The main explanations suggested in the studies point to the difference in profit orientation and the motivation of employees as key factors for explaining this. However, more research should be devoted to explaining these observations, based on the differences in the structure, operational practices and historical role of not-for-profits in specific institutional contexts.

Another theoretical point (usually not addressed clearly) in comparative public–private provider studies is that the political reasons for using private actors can vary significantly and that this is likely to have impact on the results. Contracting out can be done for purely ideological purposes. It may be done to save costs, to increase the service and quality or to boost a market and promote the development of private enterprise. This means that the use of private actors can be successful from some perspectives but not from others.

An important observation from the present review is that many studies that addressed the economic effects of ownership failed to account for quality and operational differences, such as patient selection, although this is potentially very important for the economic results. This represents an important barrier for cross-study comparison, as the tendencies regarding economic performance may be associated with different outcomes in different studies and contexts. An underlying reason for this observation is the challenge of measuring quality consistently. The literature distinguishes among input, process and outcome quality. Many studies focused on the two first dimensions as proxies for the overall quality, as it is easier to obtain data on these issues. However, the real test of benefits to patients lies in the outcome quality. There are extensive efforts to improve the collection of such data in many countries, but this effort has not yet been sufficiently integrated in efficiency studies.

In addition to the theoretically based explanations, there may be specific methodological explanations for the diverse results. Shen et al. [46] investigated such issues (also [23]. They found that variation in the direction and size of ownership effects can be explained by differences in research focus and methodology as described above.

Another methodological issue is that the number of studies and underlying cases included in this scoping review may be insufficient to show clear patterns. This argument is somewhat contradicted by the fact that this study can be seen as an extension of previous review studies, which also tended to show mixed results with a slight tendency to favor public and PNFP organizations as shown above.

Overall, it seems fair to conclude that contextual circumstances can be at least as important as ownership. Furthermore, that we need more systematic analysis of the dimensions of the context in order to find patterns in the relationship between contextual circumstances and performance for public and private providers.

## Conclusion

This paper investigated whether there is evidence that private delivery organizations perform better than public delivery organizations in European healthcare systems. This topic was studied using a scoping review of the available evidence from recent studies conducted within the European region. We identified 24 studies that reported economic efficiency measures or quality in their comparison of hospital organizations with different ownership forms. The studies covered a wide range or European countries, including Austria, Germany, England, France, Greece, Italy, Spain, Switzerland and Norway. The majority of the studies (*n* = 17) found in the database searches addressed the economic performance of public and private specialized care organizations. Seven studies addressed quality.

In terms of economic performance, most studies focused on technical efficiency using DEA or SFA techniques. Fifteen studies compared PUB hospitals to PFP hospitals. Some studies reported technical, cost and profit efficiency (see Table 3). About half of these studies reported that public hospitals are superior to PFP hospitals in efficiency. Most of the other studies found insignificant differences. Only one study reported that PFP hospitals have better profit efficiency. Eight studies compared the performance of PFP hospitals and PNFP hospitals. The majority of these studies found that PNFP hospitals are superior in terms of technical, cost and profit efficiency. Only one study pointed to responsiveness as a performance measure where PFP hospitals are better than PNFP hospitals. Finally, we found 11 studies compared PUB hospitals and PNFP hospitals. Most of these studies reported insignificant differences. In the remaining studies, we found slightly more studies presented PUB hospitals as superior to PNFP hospitals.

Summing up, our review of 17 studies representing more than 5500 hospitals across Europe showed that public hospitals are most frequently reported as having the best economic performance compared to PNFP and PFP hospitals. PNFP hospitals are second, while PFP hospitals are least frequently reported as superior. However, a sizeable number of studies did not find significant differences. In terms of quality, the results were mixed, and it is not possible to draw clear conclusions about the superiority of an ownership type. A few studies analyzed patient selection. They indicated that public hospitals tend to treat patients who are slightly older and have lower socioeconomic status, riskier lifestyles and higher levels of co-morbidity and complications than patients in private hospitals.

This scoping review pointed out shortcomings in the available studies, and future studies are needed to investigate the relationship between contextual circumstances and performance. A significant weakness in many studies was the failure to account for quality, patient selection and other operational dimensions, which may have influenced the results. This weakness should also be addressed in future comparative studies.

## Abbreviations

ALoS: Average length of stay
ASA: The American Society of Anesthesiologists
BMI: Body mass index
COLS: Corrected ordinary least squares
DEA: Data envelopment analysis
DRG: Diagnosis related group
PCI: Percutaneous coronary intervention
PD: Probability of default
PFP: Private for-profit
PNFP: Private not-for-profit
PPP: Public–private partnership
RCT: Randomized controlled trial
SD: Standard deviation
SFA: Stochastic frontiers analysis
SHI: Social Health Insurance
STC: Specialist treatment center
